# Effects of Polybrominated Diphenyl Ethers on Child Cognitive, Behavioral, and Motor Development

**DOI:** 10.3390/ijerph15081636

**Published:** 2018-08-02

**Authors:** Elizabeth A. Gibson, Eva Laura Siegel, Folake Eniola, Julie Beth Herbstman, Pam Factor-Litvak

**Affiliations:** 1 Department of Environmental Health Sciences, Mailman School of Public Health, Columbia University, New York, NY 10032, USA; eag2186@cumc.columbia.edu (E.A.G.); jh2678@cumc.columbia.edu (J.B.H.); 2 Department of Epidemiology, Mailman School of Public Health, Columbia University, New York, NY 10032, USA; els2205@cumc.columbia.edu (E.L.S.); fe2113@cumc.columbia.edu (F.E.)

**Keywords:** polybrominated diphenyl ethers (PBDEs), child neurodevelopment, child cognition, child behavior, child motor development, review

## Abstract

Polybrominated Diphenyl Ether (PBDE) flame retardants are environmental chemicals that cross the placenta during pregnancy and have shown evidence of neurotoxicity. As the in utero period is a sensitive developmental window, such exposure may result in adverse childhood outcomes. Associations between in utero PBDE exposure and neurodevelopment are found in animal models and increasingly in human population studies. Here, we review the epidemiological evidence of the association between prenatal exposure to PBDEs and motor, cognitive, and behavioral development in infants and children. Published work suggests a negative association between PBDE concentrations and neurodevelopment despite varying PBDE congeners measured, bio-specimen matrix used, timing of the biological sampling, geographic location of study population, specific developmental tests used, age of children at time of testing, and statistical methodologies. This review includes 16 published studies that measured PBDE exposure in maternal blood during pregnancy or in cord blood at delivery and performed validated motor, cognitive, and/or behavioral testing at one or more time during childhood. We evaluate possible mediation through PBDE-induced perturbations in thyroid function and effect measure modification by child sex. While the majority of studies support an adverse association between PBDEs and neurodevelopment, additional research is required to understand the mechanism of action, possibly through the perturbations in thyroid function either in the pregnant woman or in the child, and the role of biologically relevant effect modifiers such as sex.

## 1. Introduction

Polybrominated Diphenyl Ethers (PBDEs) are a group of man-made chemical compounds with flame resistant properties that are applied to furniture, plastics, electronics, paints, textiles, and construction materials. These compounds are released into the environment, as they are not covalently bound to other materials. PBDEs are persistent organic pollutants (POPs), with lipophilic properties that allow accumulation in lipid-rich tissues. This bioaccumulation is of particular concern with regard to multiple outcomes over the life course, as PBDEs have been associated with both endocrine disruption and neurotoxicity [1,2,3,4]. While other reviews have looked at isolated neurodevelopmental domains with respect to PBDE exposure, particularly during childhood [5,6], the present review aims to provide a report of the current literature regarding the effects of prenatal PBDE exposure on neurological outcomes including motor, behavior, and cognition in children, as well as examining mediation by maternal and child thyroid hormone, effect measure modification by sex, and the causal framework underlying these reported associations.

Individual PBDE congeners are categorized according to the number and position of their bromine atoms. Of the total 209 possible PBDE congeners, three groupings are found in commercial products: penta-BDEs, octa-BDEs, and deca-BDEs. Current commercial use of these congener groupings varies considerably as penta- and octa-BDEs were banned in Europe in 2004. In the U.S., production of penta- and octa-PBDEs voluntarily ceased in 2004. In Europe, deca-BDEs were banned from electrical equipment and electronics in 2006 but continue to be used in other products; in the U.S. a gradual phasing out of deca-BDEs ended in 2013. Asia and other parts of the world have widely varying regulations of penta-, octa-, and deca-BDEs. In the U.S., PBDE concentrations measured in polyurethane foam have decreased since the phase-out in 2004 [7], as have concentrations measured in child blood samples [8]. However, due to their persistence in the environment, PBDE exposure remains through products that are infrequently replaced.

Detectable concentrations of PBDE congeners are found in air, water, sediment, and animals worldwide; species higher on the food chain generally have higher concentrations in biological tissues. The half-life of PBDEs varies depending both on the congener and on the substance in which it is found [9], with total body burden half-lives of 1.8 years for BDE-47, 2.9 years for BDE-99, 1.6 years for BDE-100, and 6.5 years for BDE-153 [10]. Due to the relatively long half-lives, the accumulation in adipose tissue, and the greater concentration in species atop the food chain, the possible harmful associations between PBDEs and human health outcomes persist, despite being phased-out of commercial use. Measurement of PBDEs in the U.S. National Health and Nutrition Examination Survey (NHANES) show lower average concentrations in blood levels post-phase-out (2007–2008), when compared to pre-phase-out (2003–2004), but the confidence intervals around the means largely overlapped [11].

PBDEs and thyroxine (T4), the primary circulatory thyroid hormone, are similar in stereochemical structure. T4 is a primary regulator of fetal development and of metabolic regulation throughout the life course. In vitro evidence suggests that PBDEs may disrupt thyroid hormone production by binding to thyroid hormone receptors [12], and animal models have shown direct effects of PBDEs on the thyroid gland [13,14]. As human gestation is a critical period in brain development, and maternal thyroid hormone is crucial during this time for brain development [15,16], disruption of maternal thyroid hormone may be deleterious.

Epidemiological studies find high variability in exposure to PBDEs across geographic locations on a global scale [17]. This variability complicates the ability to detect associations between the exposure and outcome in study samples sourced from populations with generally low exposure levels relative to more highly-exposed populations (as the dose response relationships may not be linear); however, within-study associations, compared to between studies, allow for the construction of a broader picture regarding the dose-response relationship. The persistence of these chemicals in the environment necessitates a close revisit of the current evidence to inform future research that may be conducted to advance our understanding of how and at what levels prenatal PBDE exposure affects neurodevelopment.

## 2. Methods

The aim of this review is to answer the question of whether exposure to PBDEs in utero negatively impacts cognitive, motor, and behavioral neurodevelopment in children. This serves as an update to a 2014 review which considered these three broad neurodevelopmental domains and included eight studies [18]. Two other reviews on pre- and postnatal PBDE exposure published in 2017 focused on specific neurological endpoints, IQ/ADHD and child behavior [5,6]. The current review updates the existing literature by including more recent studies, concentrating on prenatal exposure, and investigating neurodevelopment collectively, including motor development, which was not included in the more recent reviews. We additionally ask whether previous studies considered perturbations in thyroid hormone as a possible mechanism for the observed associations. We performed a comprehensive literature search and selection process, a critical assessment and synthesis of the data, subgroup analyses, and final preparation of the review document [19].

A literature search was performed using the electronic databases PubMed and Web of Science. Only studies published in English before May 2018 were eligible for inclusion. The search was operationalized by focusing on epidemiologic evidence of maternal exposure to PBDEs and childhood neurodevelopment. The exposure search term was defined as: Polybrominated Diphenyl Ethers; the outcome search term was defined as: Neurodevelopment. We expanded our search by considering references included in articles found using these search terms.

**Figure 1:**
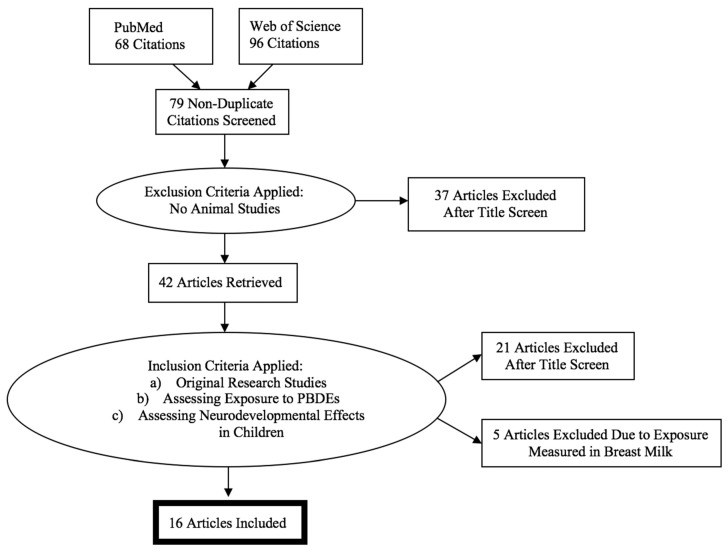
Selection process for systematic review.

Studies were excluded if they did not contain human subjects. The inclusion criteria were: (a) original research, (b) exposure to PBDEs measured in maternal serum drawn during pregnancy or from cord blood at birth (PBDEs are lipophilic and easily cross the placenta, though placental transport of PBDEs is dependent on chemical structure [20], with high correlation between matched maternal and cord blood (*r* = 0.7, *p* < 0.01) and higher concentrations in cord blood than maternal samples (*p* < 0.01) reported [21]), and (c) neurodevelopmental (i.e., cognitive, behavioral, or motor developmental) effects in children at any age. We excluded reviews, abstracts, case studies, and commentaries, as well as those that did not collect biomarkers on an individual level. After a final full-text examination, five studies that only measured PBDEs in breast milk were also excluded given that the focus of this review is prenatal exposure. The selection process is shown in Figure 1, and after applying the inclusion criteria, 16 studies were included in this review.

We assessed internal validity with a modified version of the eight-item Newcastle-Ottawa Scale for Assessing the Quality of Nonrandomized Studies [22]. The modification of the scale is included in the Appendix A (Table A1).

Below, we first review the studies included in the analysis, with special attention to the exposure measurement and levels and the control variables, and we review each outcome—i.e., cognition, behavior and motor function—separately. We also review whether the authors examined effect modification or mediation by maternal thyroid concentration and child sex.

## 3. Summary of Studies

Sixteen studies assessed the effect of prenatal exposure to PBDEs on child neurodevelopment. All analyses were derived from longitudinal birth cohorts—three from Europe (France, Spain, and the Netherlands [23,24,25]); three from Asia (China, Taiwan, and Korea [26,27,28]); and three from North America. The North American birth cohorts were: CHAMACOS in Salinas Valley, California [29,30], HOME in Cincinnati, Ohio [31,32,33,34,35,36], and World Trade Center in New York, New York [37,38]. A summary of the studies can be found in Table 1.

**Figure 2:**
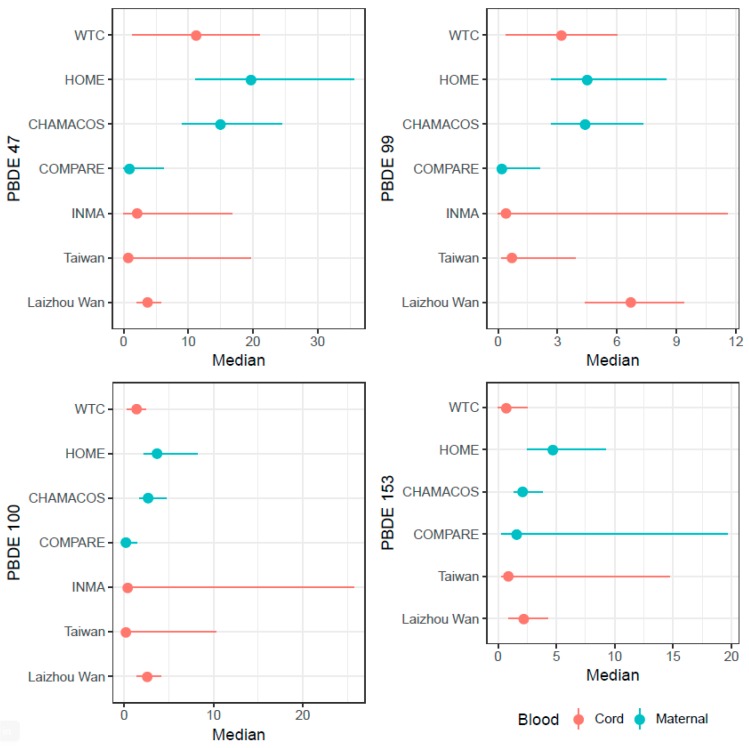
PBDE concentrations of BDE-47, -99, -100, and -153 across cohorts. 1. Points represent PBDE concentration medians. Ranges represent 25th to 75th percentiles for World Trade Center (WTC), Health Outcomes and Measurements of the Environment Study (HOME), Center for the Health Assessment of Mothers and Children of Salina (CHAMACOS), and the Chinese (Laizhou Wan) cohort. Ranges represent minimum and maximum for the Taiwanese and Spanish (INMA) cohorts.

Sample sizes ranged from 36 mothers in the Taiwanese cohort [27] to 622 in CHAMACOS [30]. The studies tested for a range of exposures to different congeners, but the present review focuses on BDE-47, -99, -100, -153, and the sum of the concentrations of these congeners as they are most consistently found in the environment and in human blood samples [11] (Figure 2). The year of exposure measurement varied from 1997 in INMA [24] to 2012 in Laizhou Wan [26]. Six of the studies (in the WTC, CHAMACOS, INMA, and COMPARE cohorts) measured exposure prior to 2004 [23,24,29,30,37,38], the year when penta- and octa-BDEs were phased out or banned in their respective locations, and seven studies (in the HOME and PELAGIE cohorts) measured exposure between 2002 and 2006 and included the year of congener removal in the enrollment window [31,32,33,34,35,36]. The studies in Asia were conducted in 2009 [27], 2011–2012 [28], and 2012 [26], but no regulation currently exists for PBDE exposure in either China or Korea [39], and Taiwanese regulation did not take effect until 2016. Depending on the study, prenatal PBDE exposure was measured in either maternal and/or cord blood. Dyads were followed from birth through between one and twelve years of age. Results were reported in terms of a shift in the population distribution of outcome scores, using estimated betas from linear regression based on one or multiple measures of exposure and outcome in HOME, PELAGIE, CHAMACOS, WTC, CHECK, and the Laizhou Wan cohorts [25,26,28,29,30,31,32,33,34,35,36,38], and in terms of group odds or risk of outcomes, using odds ratios in HOME, CHAMACOS, and the Taiwanese cohort [27,29,31], risk ratios in INMA [24], and incidence rate ratios in WTC [37]. One study reported correlations between exposure and outcomes [23].

## 4. Results

### 4.1. Motor

Nine studies measured motor development in children exposed prenatally to PBDEs on multiple scales across studies. These validated scales measure different aspects of motor development and are targeted at specific ages to measure age-appropriate development (See Table 2). Chen et al., Ding et al., Herbstman et al., Eskenazi et al., Sagiv et al., and Braun et al. evaluated associations between PBDEs and motor development in terms of number of points lost relative to increased PBDE exposure. Shy et al. and Gascon et al. assessed motor development in terms of relative odds between those with high and low exposure to PBDEs and relative risk between those characterized as exposed (>Limit of Quantification (LOQ)) to PBDEs compared to those unexposed (<LOQ) to PBDEs. Roze et al. used adjusted correlations to measure relationships between PBDE exposure and motor development. Across studies with outcome measurement ranging from 1 to 10 ½ years of age, the majority observed null results or negative associations between PBDE exposure and child motor development.

Herbstman et al. observed a significant association between BDE-100 and lower psychomotor development at one year [38]. Eskenazi et al. found that maternal ΣPBDEs were significantly associated with poor fine motor dexterity at five and seven years of age and with finger taps at age five [29]. Gascon et al., Shy et al., Chen et al., Ding et al., and Sagiv et al. detected no significant associations with motor function at ages four, five, one and two, or nine and twelve years, respectively [24,26,27,30,31].

### 4.2. Cognitive

Cognitive development assessment focused on executive function, working memory, and IQ (full scale, verbal, and performance) and was evaluated in 13 studies. These studies employed validated tests to measure different aspects of age-specific cognitive development (See Table 2). Chen et al., Chevrier et al., Eskenazi et al., Ding et al., Herbstman et al., Sagiv et al., Vuong et al. (2016), Vuong et al. (2017b), Braun et al., and Zhang et al. evaluated associations between PBDEs and cognitive development outcomes in terms of number of IQ or test points lost. Shy et al., Vuong et al. (2016), Gascon et al., and Chen et al. examined cognitive outcomes in terms of relative odds and risks, while Roze reported adjusted correlations between exposure and outcome. Across studies of cognitive development in children between 1 and 12 years of age, PBDE exposure was repeatedly associated with decreased cognitive functioning, though not all age/test measures were statistically significant.

**Figure 3:**
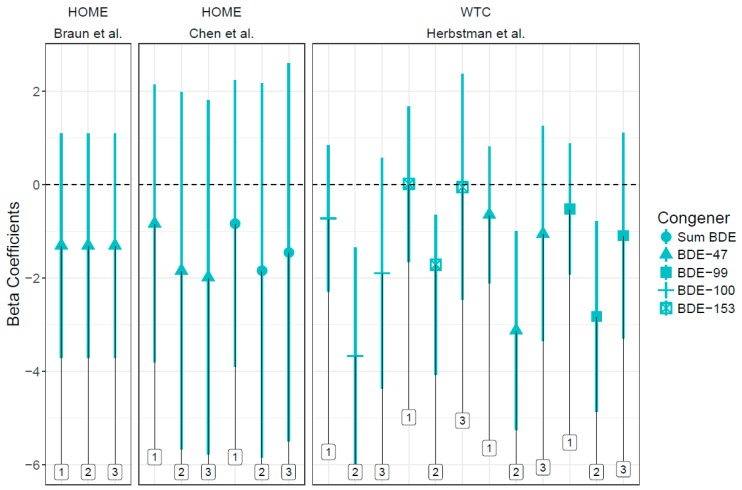
Associations between PBDEs and Bayley Scales coefficients and 95% confidence intervals across studies. 1. Points indicate beta coefficients (with 95% confidence intervals) for PBDE congeners’ association with Bayley Scale Mental Development Index at ages 1, 2, and 3 in the WTC and HOME cohorts. Age is noted in labels above. Negative coefficients indicate decreased neurocognitive functioning.

**Figure 4:**
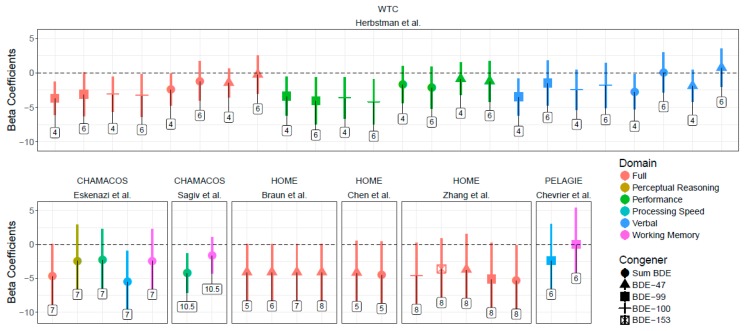
Associations between PBDEs and WISC scores and 95% confidence intervals across studies. 1. Points indicate beta coefficients (with 95% confidence intervals) for PBDE congeners’ association with Wechsler Score (Full Scale, Verbal, Performance, Working Memory, Perceptive Reasoning, and Processing Speed) at ages between 1 and 10.5 in the WTC, HOME, PELAGIE, and CHAMACOS cohorts. Age is noted in labels above. Negative coefficients indicate decreased neurocognitive functioning.

In addition to studies using the Wechsler Intelligence Scales for Children (WISC) and Preschool and Primary Scales of Intelligence (WPPSI) and Bayley Scales of Infant Development (BSID) (See Figure 3 and Figure 4), Ding et al. detected a decrease in language domain developmental quotients in children with higher prenatal exposure to BDE-99 [26]. Zhang et al. identified a negative association between ΣPBDEs and Reading Composite score and full-scale IQ and between BDE-100 and BDE-153 and Reading Composite score at age eight [33]. Shy et al. found increased odds of cognitive disability in the high ΣPBDE exposure and the high BDE-99 exposure groups (both compared to low exposure), which contradicts the hypothesized direction of association [27]. Vuong et al. observed higher odds of having a low score for global executive functioning, with increased BDE-153 [32]. Gascon et al. did not find an increased risk between prenatal PBDE exposure and cognitive measures [24].

### 4.3. Behavior

Studies evaluated associations between prenatal PBDE exposure and child behavioral development assessed at multiple time points using multiple scales across studies (Table 2). Outcome measures focused on attention, hyperactivity, impulsivity, social competence, internalizing problems (emotional reactivity, anxious/depressed scales, somatic complaints, withdrawn behavior), externalizing problems (attention problems and aggressive behavior), and ADHD. Twelve studies investigated associations between prenatal exposure to PBDEs and behavior in childhood. Eskenazi et al., Chen et al., Ding et al., Sagiv et al., Vuong et al. (2016), Zhang et al., Vuong et al. (2017a), and Braun et al. measured outcomes on a population scale as number of points lost. Shy et al., Gascon et al., Cowell et al., and Chen et al. examined behavioral outcomes in terms of relative odds, risks, and rates. Roze et al. evaluated adjusted correlations between PBDE exposure and behavioral domains. In studies of behavioral outcomes in children between 2 and 12 years old, PBDE exposure was consistently associated with decreased behavioral neurodevelopment or increased behavioral problems, though again, not all age/test measures were statistically significant.

**Figure 5:**
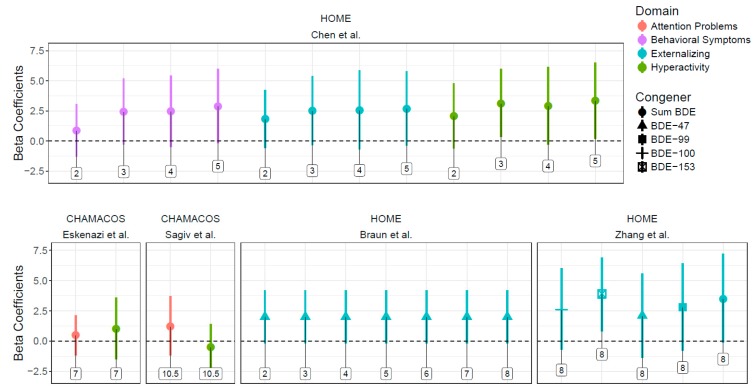
Associations between PBDEs and BASC scores and 95% confidence intervals across studies. 1 Points indicate beta coefficients (with 95% confidence intervals) for PBDE congeners’ association with Behavioral Assessment System for Children between ages 2 and 10.5 in the CHAMACOS and HOME cohorts. Age is noted in labels above. Positive coefficients indicate increased behavioral problems.

In addition to studies using the Behavioral Assessment System for Children (BASC) (See Figure 5), studies used various other validated tests. Eskenazi et al. observed associations between ΣPBDEs and increased errors of omission and ADHD confidence index at age five, and with increased total DSM-IV (inattention, hyperactivity, and impulsiveness) scale, increased inattentive subscale, and increased ADHD index at age seven [29]. Ding et al. found the increased BDE-47 was associated with a decrease in social domain developmental quotient in children at age two [26]. Sagiv et al. found consistently poorer attention in children with higher prenatal exposure to PBDEs; decreased processing speed (an indicator of attention) at ten and a half years and increased hit rate standard error by block (an indicator of performance inconsistency, a symptom of ADHD) and errors of omission at nine and twelve years were negatively associated with ΣPBDEs [30]. Vuong et al. identified an association between increased ΣPBDEs, BDE-99, and -153 and poorer behavior regulation [32]. Shy et al. identified associations between BDE-99 and ΣPBDEs and worse adaptive behavior [27]. Cowell et al. observed a significant incidence rate ratio between both BDE-47 and -153 exposure and attention problems at age four [37]. Roze et al. found significant associations between BDE-99 and improved total behavior and internalizing behavior, contradicting the hypothesized direction of association; they also detected correlation between increased BDE-47 and -99 and decreased sustained attention [23].

### 4.4. Interaction and Mediation

**Figure 6:**
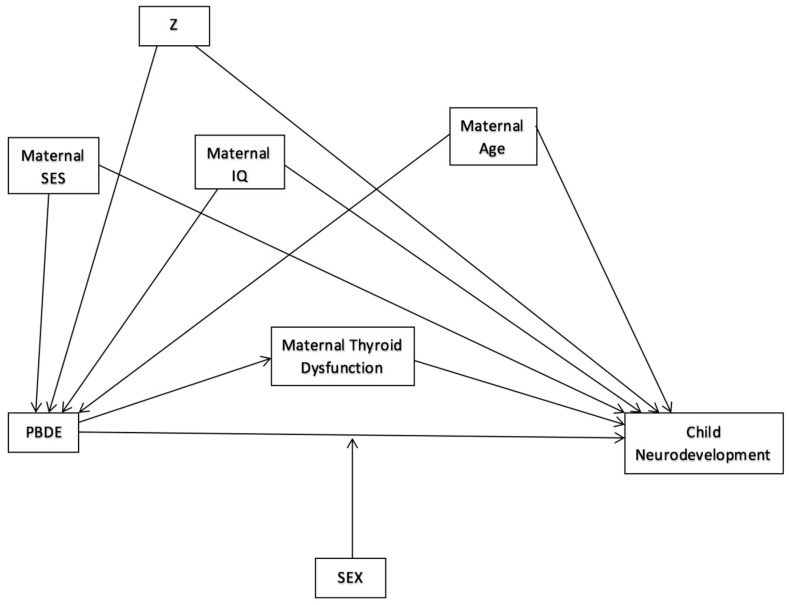
Hypothesized Directed Acyclic Graph of PBDE exposure and neurodevelopment. 1. DAG shows theoretical direct effect of PBDEs on child neurodevelopment, possibly mediated by maternal thyroid dysfunction, confounded by maternal SES, age, and IQ, and any possible “Z” confounders, and modified by child sex.

Thyroid function is a possible mediator of PBDE exposure on motor, cognitive, and/or behavioral outcomes (See Figure 6). Although thyroid hormones were measured in three of the nine cohorts evaluated [23,24,29], thyroid hormones were only assessed as a mediator in one study. In that study, inclusion of maternal T4 or TSH did not alter the relationship between PBDEs and neurobehavioral development [29]. Given the lack of studies assessing mediation by thyroid hormone, there is not adequate evidence to refute or confirm the hypothesis of mediation through the thyroid pathway.

The associations between purported endocrine disruptors and neurodevelopmental outcomes are posited to differ by sex (See Figure 6). Nine studies assessed interaction between prenatal PBDE exposure and infant or child sex. Gascon et al. reported higher levels of BDE-47 in female cord blood than in that of males but did not assess effect measure modification by sex [24]. Chen et al., Cowell et al., Sagiv et al., Vuong et al. (2017a), and Kim et al. investigated but found no indication of effect modification [28,30,31,35,37]. Sagiv et al. and Vuong et al. (2017a) did find signs of effect measure modification by sex of the association between childhood PBDE exposure and neurodevelopment.

Eskenazi et al. detected modification by sex of PBDEs on motor function—the relationship in five-year-olds was predominantly in males, and in seven-year-olds, females [29]. Chevrier et al. did not find an association between prenatal PBDE exposure and cognitive function, and thus did not find effect measure modification of the association. They did, however, find males more susceptible than females to the adverse impact of PBDE exposure through household dust on verbal comprehension [25]. The first study by Vuong et al. identified effect measure modification by sex of the relationship between in utero PBDE exposure and neurodevelopmental outcomes. Increased BDE-153 was associated with poor behavior regulation and poor executive function in males but not in females [32]. A second study by Vuong et al. found effect measure modification of the association between four PBDEs (BDE-28, -47, 99, and -100) and memory retention, with better memory in males and worse memory or null results in females [36].

## 5. Discussion

Although most of the reviewed studies suggest an association between PBDE exposure and neurodevelopmental outcomes in children, the strength of the reported association varies widely both between and within studies depending on the age at assessment and the test used to measure the outcome considered. There are several concerns regarding both the internal and external validity of these studies that, while not entirely undermining the observed associations, suggest the need for further exploration before definitive conclusions may be reached.

**Figure A1:**
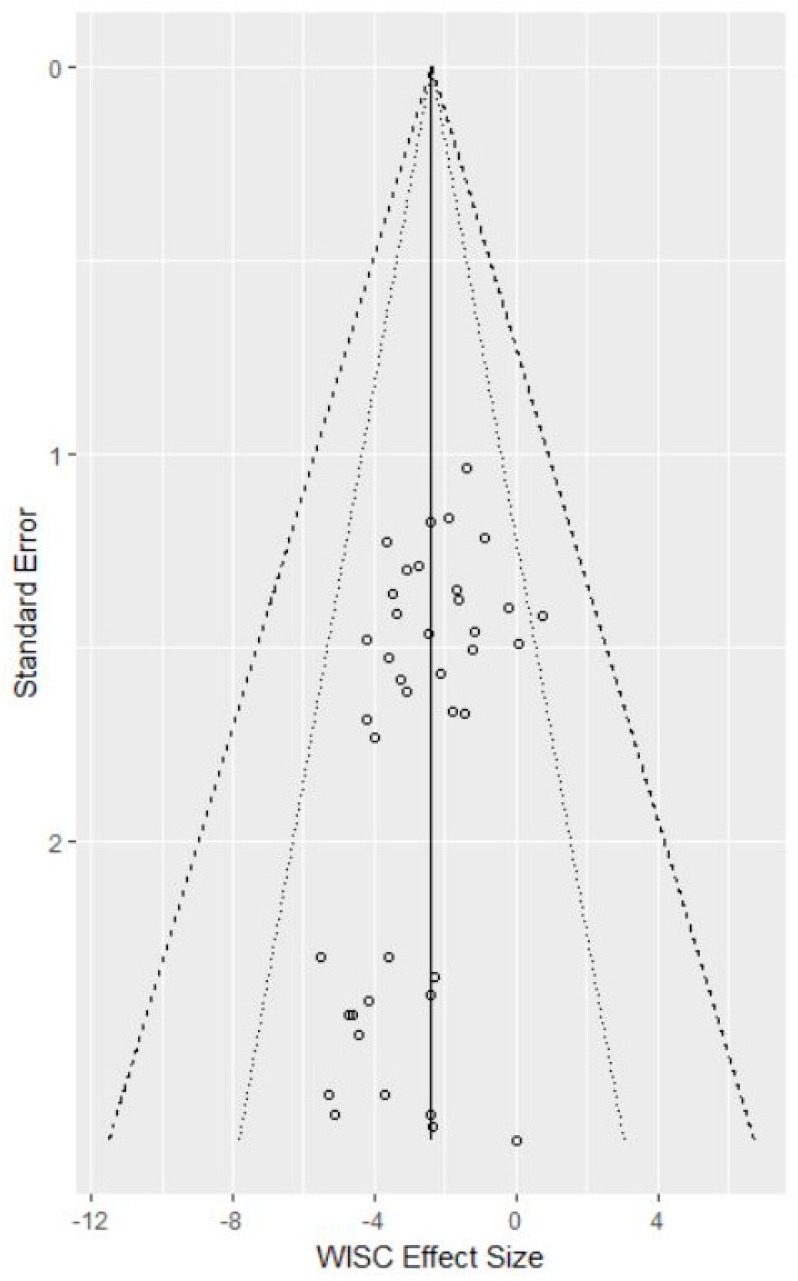
Funnel Plot of Beta Coefficients associated with Wechsler Scales. 1. Points indicate effect sizes of PBDEs on Wechsler Scale domains (Total, Performance Intelligence, Verbal Comprehension, Perceptual Reasoning, Working Memory, and Processing Speed). Standard errors, when not reported in the study, were back-calculated from the 95% confidence intervals.

The risk of bias among these cohort studies is focused on participant selection, measurement of outcomes and confounders, and possibly Type I error (See Appendix Figure A1). Temporality is not of great concern in longitudinal birth cohort studies, particularly when the exposure of interest is prenatal and the outcome is postnatal. In the present study cohorts, exposure assessment was considered adequate due to the use of biomarkers in the form of maternal and cord blood. Neurological, motor, and cognitive abilities were tested examining a large range of indicators, and the diagnostic tools utilized to examine these indicators, though all validated, were highly variable, making them internally valid, but difficult to compare across studies.

In Figure 2, the range of exposure of the most tested-for congeners can be seen. These differences in exposure level makes generalizability from one population to another difficult, though it does not affect inference within study, while explaining some of the variation in the strength of associations reported. Because some studies assessed relationships on a continuous scale by looking at population distribution and other studies assessed them using a categorical measure of exposure, between-study comparability of associations is challenging. While the dichotomization of outcomes on neurodevelopmental tests may be useful statistically, it does not allow for the examination of dose-response relationships nor for the detection of shifts in population distribution of outcomes; further, it can be the case that cut points in non-diagnostic tests may have little clinical significance. Additionally, the statistical modelling methods employed in all studies included assume a linear relationship between PBDE exposure (at times categorized or log-transformed) and neurodevelopmental outcome (whether with an identity, logit, or negative binomial link function), possibly leading to model misspecification if true exposure-response curves are nonlinear. As a potential mechanism of PBDE action on neurodevelopment is through hormone disruption and hormones are known to have nonmonotonic responses, we cannot ignore the potential for nonlinear associations.

Most studies adjusted for similar covariates (see Table 3), and our own directed acyclic graph (see Figure 6), while more parsimonious than many of the multivariable models employed in the studies, confirms the a priori logic of the included covariates. The quality of covariate assessment varied between studies and sometimes was not clearly defined.

Environmental exposures, including known neurotoxicants such as polychlorinated biphenyls (PCBs), lead, mercury, organophosphate pesticides, and environmental tobacco smoke, could potentially be correlated with PBDE exposure and with child neurodevelopment, thus confounding the hypothesized relationship. Organochlorine compounds including PCBs may share exposure routes and effects due to structural similarity with PBDEs [24], making it important to clarify associations observed between PBDEs and neurodevelopment while controlling for PCB co-exposure. Roze et al. and Zhang et al. both measured PCBs within the same study but did not report correlations between the two toxicants or adjust for PCBs in statistical models. Herbstman et al. did not include PCBs in their analyses, but due to residual effects of the World Trade Center attacks, participants were likely exposed to both. They included a proxy measurement for indoor particulate matter, cord blood lead, and mercury as additional environmental exposures [38]. Eskenazi et al. and Vuong et al. (2016) both adjusted for PCBs in sensitivity analyses and found that they did not confound the observed relationships. One study adjusted for PCB-153 in all analyses [25]; and only one presented the correlation between PBDEs and PCBs (PBDE-47 with ΣPCBs = 0.25, *p* < 0.05) [24].

In addition, we assessed the possibility of bias in the present studies from co-pollutant confounding due to chemical mixtures. There is a high degree of collinearity between both individual PBDE congeners as well as PBDEs and other chemical substances (Woodruff et al., 2011). Chemical exposures are often not isolated events, and populations that are likely to have high levels of exposure to PBDEs are also likely to have high levels of exposure to other chemicals. Studies that did not account for other chemicals were considered to be at high risk of bias. At the same time, the main effect remained in those studies that controlled for other chemical exposures [29,30]. While we know that including additional chemical exposures with a common (but unmeasured) source may lead to the amplification of bias due to residual confounding [40], a strong causal link between co-pollutants and the outcome favors including the co-pollutant in the model. This argues for the inclusion of known neurotoxins (e.g., lead, PCBs) that may be correlated with PBDE exposure.

As is often the case with longitudinal birth cohorts, both participant selection and retention were cause for concern in the included studies. In many cases, selection from the overall cohort was based on participants with available data or biosamples or those who met the study criteria at the time, not necessarily dictated by complete loss to follow up. It is possible that differential selection within the larger cohort may underestimate the true association. If those more likely to be left out of the study are also those with the highest exposures—as is often the case given that social vulnerability tends to coincide with higher rates of chemical exposures and higher dropout rates in studies—the study loses precisely those participants who would potentially demonstrate a stronger association. On the other hand, studies with more stringent inclusion/exclusion criteria that minimized loss to follow up can only be generalized to populations with similar exposure profiles.

One of the greatest concerns with the studies evaluated was the large number of statistical tests performed without adjustment. Type I error is a defined proportion; with an a priori alpha of 0.05, one out of every twenty tests performed will be erroneously significant, meaning that the more hypotheses tested, the greater number of significant results will be found by chance alone. Initially stated hypotheses and consistency in magnitude and direction of beta coefficients do help to alleviate concerns, but no studies included employed statistical adjustment for multiple comparisons. Another statistical concern in these cohorts is low power. Small sample sizes limited the ability to detect significant associations, especially concerning effect measure modification and mediation.

Studies that evaluated PBDE exposure solely through breast milk were excluded from this review. While breast milk does reflect prenatal exposure, it cannot be separated from postnatal exposure. Across studies that measured PBDEs in breast milk, similar, though weaker, conclusions were made. In the Taiwanese cohort included in this review, breast milk concentrations of PBDEs within one month of delivery were negatively associated with child cognitive scores between 8 and 12 months [41]. A cohort in North Carolina found increased externalizing behavior, specifically activity/impulsivity behavior at 36 months in association with higher PBDE concentrations in breast milk measured three months postpartum [42]. In the same cohort, PBDE concentrations were associated with anxious behavior and increased withdrawal in children at 36 months but also with improved adaptive and cognitive skills [43]. In the Spanish cohort included in this review, increased PBDE concentrations in the first breast milk sample after birth were associated with decreased MDI scores in infants between 12 and 18 months [44]. It should be noted that negative associations between PBDE exposure in breast milk and child neurocognitive outcomes are likely underestimated given the protective effect of breastfeeding on child neurodevelopment.

With varied associations between PBDE concentrations and motor, cognitive, and behavioral outcomes dependent on sex across cohorts, results suggest effect measure modification by sex. The inconsistencies across ages (greater association between PBDEs and outcomes in girls at some ages and in boys at other) may be attributed to differences in hormone levels and developmental speeds between sexes; they may also be due to post-natal exposure. Given the sample sizes in these studies, most were likely underpowered to observe different associations between PBDEs and neurodevelopment in males and females. On the other hand, observed effect modification may be a spurious finding, given the small sample sizes.

Two reviews of PBDE exposure and child neurodevelopment have been recently published. A systematic review and meta-analysis investigated a more attenuated set of neurodevelopmental outcomes [5], focusing on cognition and ADHD or attention-related behavioral conditions in children, with exposure measured in breast milk and child blood in addition to maternal and cord blood. Lam et al. found “sufficient evidence of toxicity” from PBDEs associated with child intelligence and “limited evidence of toxicity” in relation to ADHD and attention. A second review by Vuong et al. focused on behavioral outcomes, including externalizing and internalizing behavior, executive function, attention, social behaviors/Autism Spectrum Disorder, and adaptive skills [6]. They looked at both prenatal and childhood PBDE exposure periods, and their review indicated that PBDE exposure in utero is associated with impaired executive function and attention in children. Our results are consistent with Lam et al. and Vuong et al. and add to the literature in several aspects: (1) an update of the current literature, including the most recent publications, (2) a wider review of neurodevelopmental outcomes, including cognitive, behavioral, and motor development (not included in other reviews), which allows a comprehensive understanding of PBDE exposure and different neurodevelopmental domains, (3) a focus on the prenatal critical period of development (other reviews did not separate pre- from postnatal exposure), (4) an assessment of effect measure modification by sex, and (5) an assessment of mediation by thyroid hormones (mentioned as a potential mechanism of action in other reviews but not assessed).

A final note is on the subject of publication bias: it is quite likely that, given that positive results are published more than null results, other studies that found no association are not represented in this review. This would result in a weaker overall association than that presently observed. Plotting the observed effect sizes and standard errors in relation to the weighted average of effect sizes across studies modeling a subset of outcomes of interest (WISC scores across all domains) indicates little bias in main effects due to publication preference (See Appendix Figure A1). However, given the small number of studies that investigated mediation by thyroid and effect measure modification by sex, it is likely that null results for these secondary analyses were not reported.

## 6. Conclusions

This systematic review of 16 published papers on the subject of PBDEs and child neurodevelopment suggests a negative association between the presence of these chemicals in maternal and cord blood and motor, behavioral, and cognitive outcomes in children. The negative association is consistent with in vitro studies, and animal studies have suggested that PBDEs are associated with learning and memory impairment and hyperactivity [3,45]. Our analysis found similar results to the most recent review of the literature on these three neurodevelopmental domains published in 2014 which only included 8 papers and provides an update to that review [18].

The longitudinal birth cohort studies included in this review demonstrate the benefits and drawbacks of this type of environmental epidemiological research. At the same time, longitudinal birth cohort studies are the closest-to-ideal design to study associations of this kind in humans. The standardization of outcome assessment in future work will facilitate study generalizability, and the universal inclusion of maternal thyroxine levels in the study of PBDEs and other endocrine-disrupting chemicals and neurodevelopment will aid in the clarification of the mechanism.

## Figures and Tables

**Table 1:** Summary of epidemiological studies by geographic region.

Study		Exposure Matrix (Sample Size)	Years of Exposure Collection	Measured Congeners	Motor	Behavior	Cognitive
**United States**							
CHAMACOS cohort							
Eskenazi et al., 2013	Salinas Valley, California	Maternal blood during pregnancy or at delivery (*n* = 279)	1999–2000	Primary exposure: ΣPBDEs (BDE-47, -99, -100, and -153)	X	X	X
Sagiv et al., 2015	Salinas Valley, California	Maternal blood during pregnancy or back-extrapolated (*n* = 622)	1999–2002; additional 9-year-olds included in 2009–2011	BDE-17, -28, -47, -66, -85, -99, -100, -153, -154, and -183	X	X	X
HOME cohort							
Chen et al., 2014	Cincinnati, Ohio	Maternal serum at 16 weeks gestation (*n* = 309)	March 2003–February 2006	BDE-17, -28, -47, -66, -85, -99, -100, -153, -154, and -183 (with focus on BDE-47)	X	X	X
Vuong et al., 2016	Cincinnati, Ohio	Maternal serum at 4 months’ pregnancy (age 5, *n* = 201)	2003–2006	BDE-17, -28, -47, -66, -85, -99, -100, -153, -154, and -183		X	X
Zhang et al., 2016	Cincinnati, Ohio	Maternal serum at 4 months’ pregnancy (*n* = 239)	2003–2006	BDE-17, -28, -47, -66, -85, -99, -100, -153, -154, and -183		X	X
Braun et al., 2017	Cincinnati, Ohio	Maternal serum (*n* = 346)	March 2003–January 2006	BDE -28, -47, -85, -99, -100, -153, -154	X	X	X
Vuong et al., 2017a	Cincinnati, Ohio	Maternal serum at enrollment (*n* = 214)	2003–2006	BDE-17, -28, -47, -66, -85, -99, -100, -153, -154, and -183		X	
Vuong et al., 2017b	Cincinnati, Ohio	Maternal serum at 16 weeks’ gestation (*n* = 199)	2003–2006	BDE-17, -28, -47, -66, -85, -99, -100, -153, -154, and -183			X
World Trade Center cohort							
Herbstman et al., 2010	New York, NY	Cord blood plasma (*n* = 152)	2001–2002; Pregnant as of 9/11	BDE-47, -99, and -100	X		X
Cowell et al., 2015	New York, NY	Cord blood plasma (age 4, *n* = 109; age 6, *n* = 107)	December 2001–June 2002	BDE-47, -85, -99, -100, -153, -154, and -183		X	
**Asia**							
Shy et al., 2011	Southern Taiwan	Cord blood (*n* = 36)	2007–2008	BDE-15, -28, -47, -49, -99, -100, -153, -154, -183, -196, and -197	X	X	X
Ding et al., 2015	Shandong Province, Northern China	Cord blood serum (12 months, *n* = 192; 24 months, *n* = 149)	September 2010–February 2012	BDE-28, -47, -85, -99, -100, -153, -154, and -183	X	X	X
Kim et al., 2018	Seoul, Anyang, Ansan, and Jeju, Korea	Maternal serum (*n* = 59)	2011–2012	19 unspecified PBDE congeners	X	X	X
**Europe**							
Chevrier et al., 2016	Brittany Region, France	Cord blood (*n* = 159); Household dust	2002–2006	BDE-47, -85, -99, -100, -119, -153, -154, -183, and -209			X
Roze et al., 2009	Northern Provinces of the Netherlands	Maternal serum at 35th week of pregnancy (*n* = 62)	October 2001–November 2002	BDE-47, -99, -100, -153, and -154	X	X	X
Gascon et al., 2011	Catalonia, Spain	Cord blood (*n* = 88)	Mid 1997	BDE-12–13, -32, -17, -28–33, -47, -100, -119, -99, -116, -85, -126, -155, -153, -183, -66, -71, -154, -138, and -190	X	X	X

**Table 2:** Associations with cognitive, behavioral, and motor development outcomes across studies.

Study			Roze et al., 2009	Herbstman et al., 2010	Gascon et al., 2011	Shy et al., 2011	Eskenazi et al., 2013	Braun et al., 2014	Chen at al., 2014	Cowell et al., 2015	Ding et al., 2015	Sagiv et al., 2015	Chevrier et al., 2016	Vuong et al., 2016	Braun et al., 2017	Vuong et al., 2017a	Vuong et al., 2017b	Zhang et al., 2017	Kim et al., 2018
Cognitive development tests	Bayley ^4^	Age		1, 2, 3		1			1–3						1–3				
		Sign ^1^		−/+, −, −		−			−						−				
	WISC ^5^	Age	5–6	4, 7			7		5			10.5	6		5–8			5, 8	
		Sign ^1^	0 ^3^	−,−	−/+			−		−			−	0 ^3^		−			−
	Other ^10^	Age	5–6		4		5				1, 2	9, 12		5, 8			8	5, 8	
		Sign ^1^	−		−		0 ^3^				0 ^3^, −	−		−, −			−/+	−, −	
Behavioral development tests	BASC^ 6^	Age					7		2–5			10.5			2–8			5, 8	
		Sign ^1,2^					−		−			−/+			−			−,−	
	Conners’ ^7^	Age					5, 7					9, 12				8			
		Sign ^1,2^					−					−				0 ^3^			
	CBCL ^8^	Age	5–6				5			3, 4, 5, 6, 7									1–2
		Sign ^1,2^	−				−			−, −, 0 ^3^, 0 ^3^, −									−
	Other ^11^	Age			4	1		4–5			1, 2	9, 12		5, 8					
		Sign ^1,2^			−	−		−/+			0 ^3^, −	−		−					
Motor development tests	Bayley ^4^	Age		1, 2, 3		1			1, 2, 3										
		Sign ^1^		−, −/+, −/+		0 ^3^			0 ^3^										
	MSCA ^9^	Age	5–6		4		5, 7												
		Sign ^1^	−		−		−												
	Other ^12^	Age	5–6				5, 7				1–2	10.5							
		Sign ^1^	−				−				0 ^3^	0 ^3^							

^1^ Sign indicates positive or negative associations with neurodevelopment. Minus (−) indicates that higher PBDE exposure was associated with poorer test scores. Plus (+) indicates that higher PBDE exposure was associated with better test scores. Combined (−/+) indicates that higher PBDE exposure was associated with both poorer and better test scores depending on the congener analyzed. ^2^ Tests for which a higher score indicates poorer performance were reverse coded so that all minus signs indicate poorer performance and all plus signs indicate better performance. ^3^ This table does not include statistical significance. Zeros indicate null effect estimates or results not presented. ^4^ Bayley = Bayley’s Scales of Infant Development, Mental Development Index (Cognitive) and Psychomotor Development Index (Motor). ^5^ WISC = Wechsler Preschool and Primary Scale of Intelligence (WPPSI-R) and Intelligence Scale for Children, Total, Verbal, Performance, and Perceptual Reasoning. ^6^ BASC = Behavioral Assessment System for Children. ^7^ Conners’ = Conners’ Kiddie Continuous Performance Test (KCP-T) and Conners’ Continuous Performance Test (CPT-II), including ADHD/DSM-IV scales, ADHD Index Scores, Inattention, Hyperactivity, and Impulsiveness Scales. ^8^ CBCL = Child Behavior Checklist. ^9^ MSCA = McCarthy Scales of Children’s Abilities, Verbal, Perceptive-Performance, Memory, and Quantitative Subscales. ^10^ Cognitive Other includes: Gesell Developmental Schedules Language Domain, MSCA, Peabody Picture Vocabulary Test, Woodcock-Johnson Test of Achievement, Developmental Neuropsychological Assessment, Wide Range Achievement Test, Rey’s Auditory Verbal Learning Test, Test of Everyday Attention for Children, Wisconsin Card Sort Task-64, Virtual Morris Water Maze, Behavioral Rating Inventory of Executive Function, Metacognition and Global Executive Function Subscales. ^11^ Behavioral Other includes: Parent-report social-emotional and adaptive behavior, Gesell Developmental Schedules Adaptive and Social Development Domains, California Preschool Social Competence Scale, ADHD Criteria of the Diagnostic and Statistical Manual of Mental Disorders, Social Responsiveness Scale, ADHD questionnaire, Developmental Neuropsychological Assessment Tower, Balloon Analogue Risk Task, Behavioral Rating Inventory of Inhibition, Shift, Emotional Control, Initiative, Working Memory, Planning, Organization of Materials, and Monitor Scales. ^12^ Motor Other includes: Gesell Developmental Schedules Motor Development Domain, Wide Range Achievement Test, Behavioral Assessment and Research Testing, Luria-Nebraska Neuropsychological Battery, Movement ABC, Touwen’s age-specific neurologic examination, and the Developmental Coordination Disorder Questionnaire.

**Table 3:** Examination of covariates included in statistical models across studies.

	Author, Year	Braun et al., 2014	Sagiv et al., 2015	Zhang et al., 2016	Vuong et al., 2016	Chen et al., 2014	Vuong et al., 2017a	Vuong et al., 2017b	Braun et al., 2017	Gascon et al., 2011	Herbstman et al., 2010	Chevrier et al., 2016	Cowell et al., 2015	Kim et al., 2018	Ding et al., 2015	Eskenazi et al., 2013	Roze et al., 2009	Shy et al., 2011
Adjusted Covariates (Confounders)	Mat. Age	√	√	√	√	√	√	√	√	√	√	√	√	√	√			√
	Gest. Age ^1^	√									√			√				
	Edu. ^1^	√	√	√	√	√		√	√	√	√	√		√	√	√		
	Race	√		√	√	√	√	√	√		√		√					
	Infant sex	√	√	√	√	√	√	√		√	√		√	√	√	√	√	
	Infant age at testing		√							√	√		√			√		
	BFD ^1^		√							√	√	√						
	SES ^1^	√	√	√	√	√	√	√	√	√				√	√		√	
	Smoking Status ^1^	√	√	√	√	√	√	√	√	√		√			√			
	Maternal IQ	√	√	√	√	√	√	√	√		√	√	√					
	Marital Status	√	√	√	√	√	√	√	√				√			√		
	Parity	√	√	√			√			√				√				√
	HOME score ^1^	√		√	√	√	√	√	√			√				√	√	
	Other chemicals ^1^	√	√	√								√						
	Total	12	11	10	9	9	9	9	8	8	8	7	6	6	5	5	3	2

^1^ Mat. Age = maternal age; Gest. Age = gestational age; Edu. = maternal education, Gest. Age = gestational age, BFD = breastfeeding duration, SES = socioeconomic status (including proxies), Smoking Status = either self-reported or by serum cotinine concentrations, HOME score = Home Observation for Measurement of the Environment, Other Chemicals include heavy metals and PCBs.

## Appendix A

**Table A1:** Evaluation on internal validity in individual studies.

	Were subjects with varying levels of exposure drawn from the same underlying population?	Was exposure assessed using validated methods?	Are we confident that the outcome of interest was not present at start of study?	Did the study adjust for confounding?	Was an independently validated measure used for outcome assessment?	Did the statistical analysis include at least 60% of the cohort over the course of the study?	Was adjustment made for multiple statistical comparisons between congeners and outcomes?
**United States**							
*CHAMACOS*							
**Eskenazi et al., 2013**	X	X	X	X	X	X (266/526 = 51%)	X
**Sagiv et al., 2015**	X	X	X	X	X	X (337/536 = 63%)	X
*HOME*							
**Chen et al., 2014**	X	X	X	X	X	X (309/389 = 79%)	X
**Vuong et al., 2016**	X	X	X	X	X	X (256/390 = 66%)	X
**Zhang et al., 2016**	X	X	X	X	X	X (239/389 = 61%)	X
**Braun et al., 2017**	X	X	X	X	X	X (229/346 = 59%)	X
**Vuong et al., 2017a**	X	X	X	X	X	X (214/390 = 55%)	X
**Vuong et al., 2017b**	X	X	X	X	X	X (199/390 = 51%)	X
*WTC*							
**Herbstman et al., 2010**	X	X	X	X	X	X (152/329 = 46%)	X
**Cowell et al., 2015**	X	X	X	X	X	X (109/329 = 33%)	X
**Asia**							
**Shy et al., 2011**	X	X	X	X	X	X (36/95 = 38%)	X
**Ding et al., 2015**	X	X	X	X	X	X (192/347 = 55%)	X
**Kim et al., 2018**	X	X	X	X	X	X (59/140 = 42%)	X
**Europe**							
**Gascon et al., 2011**	X	X	X	X	X	X (422/482 = 88%)	X
**Roze et al., 2009**	X	X	X	X	X	X (62/69 = 90%)	X
**Chevrier et al., 2016**	X	X	X	X	X	X (246/287 = 86%)	X

X indicates “Yes” or “Mostly Yes”. X indicates “Mostly No” or “No”.
